# Involvement of patient organisations in research activities: actions taken and lessons learned in a clinical research study for osteogenesis imperfecta

**DOI:** 10.1186/s13023-025-03986-9

**Published:** 2025-09-30

**Authors:** Marina Mordenti, Leonardo Panzeri, Alice Moroni, Manila Boarini, Marta Calzolari, Francesca Gurioli, Chiara Pollicini, Giulia Rogati, Alberto Leardini, Luca Sangiorgi

**Affiliations:** 1 Department of Rare Skeletal Disorders, IRCCS Istituto Ortopedico Rizzoli, Bologna, Italy; 2 Italian OI Association, As.It.O.I., Rome, Italy; 3 Movement Analysis Laboratory, IRCCS Istituto Ortopedico Rizzoli, Bologna, Italy

**Keywords:** Involvement of patient organisation, Clinical research studies, Osteogenesis imperfecta, Gait analysis, Rare diseases

## Abstract

**Background:**

Rare diseases are chronic, progressive, and debilitating conditions, affecting 3.5–5.9% of the global population. Clinical research studies are crucial for developing new diagnostic approaches and treatments and for overcoming the lack of awareness and the need of expertise surrounding these diseases. Involving patient organizations in clinical studies is widely considered a promising approach, to overcome barriers and to facilitate research activities. The aim of this paper is to present the actions taken, the relevant results, and the lessons learned from involving a patient organization in shaping, conducting, and disseminating a clinical study on rare patients with Osteogenesis Imperfecta.

**Results:**

In a context of a clinical study in which patients underwent a comprehensive, fully instrumental gait analysis and an evaluation of specific movement tasks using stereophotogrammetry and wearable sensors, we assessed all the actions taken and the results achieved by the implementation of an original collaborative model between a public institution and a national patient organization. To generalize our collaborative experience, steps and stages of the research process that can benefit the most from the support of a patient organization were identified, experimental protocol drafting, ethic committee approval, patient enrolment, and dissemination. Patients reported positive feedback in a short questionnaire on the use case experience. Moreover, we highlighted the gains and the weaknesses of this approach.

**Conclusions:**

This experience resulted in several benefits for all the actors involved, strengthening the collaboration between the PO and researchers and fostering a cohesive and cooperative network.

## Background

Rare diseases (RDs) represent a wide variety of chronic, progressive, and debilitating conditions, that are collectively characterized by low prevalence [1]. The majority of RDs have a paediatric onset and result in lifelong disability or, even, premature mortality [2, 3]. According to Nguengang Wakap, RDs affect an estimated 3.5–5.9% of the global prevalence population, which equates to approximately 300 million people worldwide [4]. This group of diseases is characterized by a lack of awareness and expertise, diagnostic delays, and limited availability of effective treatments [5, 6]. Clinical studies and research activities are essential for increasing the knowledge about RDs, shortening the long diagnostic odyssey, and promoting new approaches to treatments and therapies. Nevertheless, conducting research studies in the field of rare diseases presents unique challenges throughout the entire process, from the preparatory phase to the communication and dissemination of findings [7, 8]. Most of all, enrolling patients with RDs in research studies is a well-known and burdensome issue, due to the scarcity and geographic dispersion of patients [9]. Alternative trial designs [10, 11] were proposed, and different approaches were chosen to overcome recruitment problems in clinical trials [9]. In addition, dedicated initiatives such as the European Reference Networks (ERNs) offer valuable support in promoting and sustaining research studies [12].

Another potential source of support in several aspects of conducting clinical studies could come from patients and patient organisations (POs), which are non-profit networks of individuals representing and advocating for patients and families affected by a specific condition or a small group of conditions [13]. At present, the role of POs in the biomedical research ecosystem has expanded beyond their traditional functions, such as raising disease awareness or promoting peer support groups, becoming acknowledged and essential actors in biomedical research and healthcare [13]. Therefore, it is crucial to recognize the vital role that POs play in supporting patients and their families. Involving patient representatives in the planning and design of the clinical research process, as well as in the dissemination activities, is widely considered a promising approach, to overcome barriers and to facilitate relevant research stages including patient recruitment [13, 14]. Besides, according to Patterson, ‘supporting or promoting research on the disease’ is the top priority for POs [15].

The collaborative approach between researchers and POs has been extensively adopted in natural history and epidemiological studies based on real-world data from disease registries and structured data collections as well as in biobanking research [8, 13, 15–17].

As part of the Italian project “Smart sensors, infrastructure and management models for the security of frail people. Acronym: 4FRAILTY” (grant: PON “Ricerca e Innovazione” 2014–2020), we have successfully developed a use case titled “Advanced solutions for the management of patients with Osteogenesis Imperfecta”. This use case aims to thoroughly investigate the effectiveness of inertial measurement unit (IMU) sensors in collecting kinematic data for monitoring patients with Osteogenesis Imperfecta (OI). OI is a rare skeletal disease characterized by bone fragility, recurrent fractures, bony deformities, short stature, joint hyperlaxity and several extra-skeletal features [18].

The IRCCS Istituto Ortopedico Rizzoli (IOR) and the Italian Patients Association of Osteogenesis Imperfecta (As.It.O.I.) have incorporated a collaborative approach from the outset, following experiences previously adopted in several pharmacological trials and research studies [19] and adapting it according to this specific use case. The present study wants to report the action taken, the relevant results and the lessons learned from involving the As.It.O.I., in shaping, conducting, and disseminating a clinical research study on OI patients.

## Methods

### The clinical research study: use case overview

The use case was conducted solely at an Italian reference centre for OI and was intended to enrol children and adult (8–60 years) patients affected by OI and capable of walking freely without assistance (“walking”) to undergo a comprehensive fully instrumental gait analysis evaluation. Kinematic data were acquired simultaneously by an 8-camera stereophotogrammetric system (Vicon Motion Capture, Oxford, UK) with a validated protocol [20, 21] and a 5 IMU-based system (Euleria Health srl, Rovereto, Italy) [22]. The primary objective of this comparative study was to evaluate the IMU-based system as a more accessible and clinically viable alternative to the current gold standard for movement analysis for monitoring disease evolution. Patients performed a series of motor tasks, including routine functional activities (e.g. walking, sitting/standing from a chair), and selective joint movement (e.g. hip flexion). The study also aimed to collect multimodal data for the Registry of Osteogenesis Imperfecta (ROI) [ClinicalTrials.gov ID: NCT04115774] and to investigate patients’ Health-related quality of life (HRQoL) via EuroQoL-5-Dimensions (EQ-5D) [23] and the subjective balance confidence in performing activities via the Activities-specific Balance Confidence Scale (ABC Scale) [24].

Additionally, a satisfaction questionnaire has been submitted to patients (or their parents) to investigate their perception on the use case experience in terms of information received, feasibility of the tasks and tiredness at the end of the evaluation in a scale from 1 (very low) to 5 (very high).

This research use case was focused on evaluating movement analysis as a non-pharmacological intervention, aiming (a) to increase the knowledge on functional deficits in OI patients, (b) to promote new approaches to study the effects of the disease, (c) to find new physiotherapeutic and rehabilitative approaches to treat OI patients, and (d) to investigate HRQoL.

### The research stages and the PO involvement

As widely described in the literature, any clinical research process consists of several standard stages, from study design to dissemination of the results; POs can play a crucial role in almost all these stages of the process [13, 25].

**Fig. 1:**
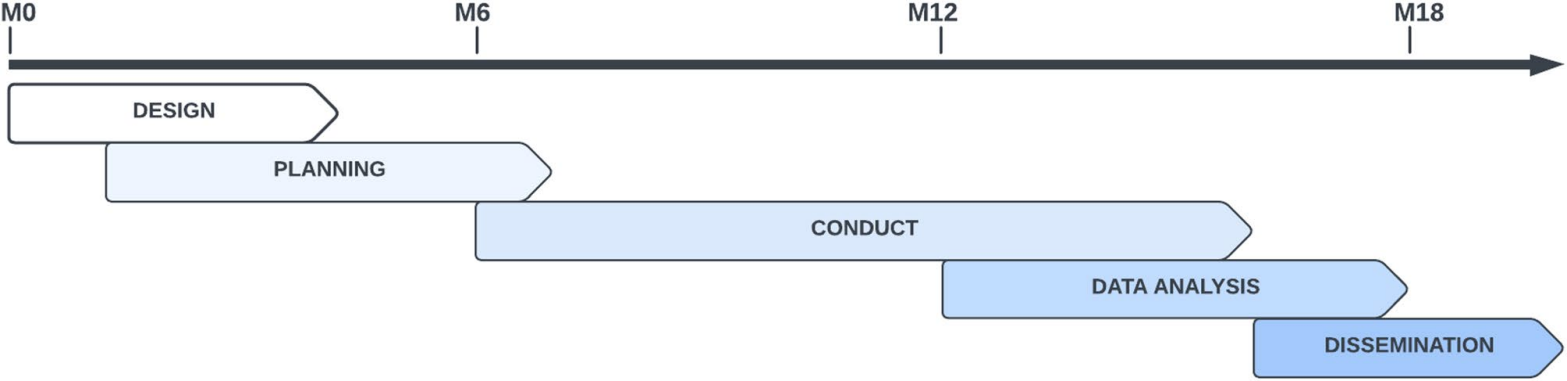
The research stages. Schematization of the 5 main stages that cover the research process adopted in the use case here conducted

Given the use case presented and its particularities, we identified the following five main stages of the research process, which are present in almost all clinical studies: design, planning, conduct, data analysis, and dissemination. The Fig. 1 presents the 5 stages and the timeline of the present study to give a clear overview of the action performed.

The entire PO involvement lasted 18 months. Each stage consists of several steps and standard activities that can benefit from the involvement of a PO, such as the definition of patient-centred endpoints or the dissemination of the results, specially to laypeople.

The involvement of POs in research studies has been extensively examined and discussed in various studies and scientific articles. Several reviews from different perspectives, including the type of frameworks, ethical challenges, and practical recommendations, have been published on this topic [25–27].

There are several compelling reasons to promote the participation of POs in research studies. According to Heaven, when designing research, it is important to take into account ethical and moral considerations, economic barriers, and practical aspects [28]. Hence, inclusivity and accessibility aspects should be always addressed, especially in RDs, as POs can provide valuable inputs in the research process, given that they can face somehow all these issues daily. Moreover, particularly in the context of RDs, the small number of affected patients presents the challenge of reaching an appropriate number to propose and to be recruited for a clinical research study. This task becomes even more challenging when there are in place strict inclusion criteria, as this may further reduce the number of eligible patients, or a short timeframe, as in the present case, because of a delay in the start of the project. Another key aspect is the dissemination of information to laypeople, which can greatly benefit from the involvement of POs. Most of these are indeed skilled in effective communication with patients and their families and can facilitate the dialogue between researchers and the public audience.

Finally, a new funding model, known as venture philanthropy, is profoundly transforming research in rare diseases. This approach enables POs to become true strategic players in clinical development, through targeted investments that help launch clinical trials even in high-risk settings and therapeutic areas that, until recently, were considered unattractive by the traditional pharmaceutical industry. The key point is that these organizations do not merely provide funding: they contribute in-depth knowledge of the disease, a network of highly engaged patients, valuable clinical data, and strong advocacy capabilities. All of this helps generate a social return, even before a financial one. However, in order to avoid conflicts of interest and maintain public trust, it is essential to ensure robust governance, full transparency, and ethical management of agreements, often formalized through complex contracts that govern aspects such as intellectual property rights, royalties, and development milestones [29].

As.It.O.I. was fully involved in the present use case, from the outset to dissemination, taking into account all relevant factors.

### The As.It.O.I.

As.It.O.I. was founded in Padua in 1984 by Marcella Zingales, a mother determined to find specialists to provide support and answers for her daughter Fabrizia, who was suffering from OI.

The association’s first members were parents of children with OI. From the beginning, groups of Italian medical doctors and physicians, willing to deal with the disease at a time when there were no in-depth studies in Italy, gathered around As.It.O.I.

As.It.O.I. was one of the founding associations of OIFE (Osteogenesis Imperfecta Federation Europe) in the early 1990s. OIFE is the umbrella organization representing various European organizations for OI.

Since its foundation, the mission of As.It.O.I. has been twofold: firstly, to bring together families and individuals affected by problems related to OI, and secondly, to raise awareness and to support medical doctors, specialists, and the scientific research on OI. This has been achieved by encouraging research activities, by supporting new therapies testing, and by making medical progress through active collaborations in various scientific projects and in various POs at European and international levels.

Over time, the association has developed skills and initiatives to answer questions of everyday life and to address issues related to the quality of life for people with OI and their families. This has been achieved through collaborations with various national and international organisations, such as EURORDIS (European Organisation for Rare Diseases) [30].

## Results

### Preliminary descriptive results on the use case

During the ten-month enrolment period, a total of 30 patients participated in the study. All patients underwent a fully instrumented gait analysis using both stereophotogrammetry and an IMU-based system. In addition, motor tasks from daily living, such as sitting and standing from a chair, as well as specific movements like hip flexion, were evaluated. According to scientific literature in rare diseases scenario [31, 32], less conventional study designs and analytic approaches may be used and studies can be performed on very small patients’ population. 

Of the entire cohort, 17 patients (56.7%) were female. Most of the patients were adults (83.3%), ranging in age from 9 to 56 years old. Type I OI was the most common (90%). This is related to the inclusion criteria, which required patients to be capable of walking without assistance, as well as the higher frequency of type I among all OI types. Nevertheless, 79% of cases presented with disease-related functional limitations or deformities of the spine, upper limbs, or lower limbs.

Twenty-two patients from the entire cohort completed the satisfaction questionnaire, with supportive results regarding the received study information (4.8/5) and the feasibility of the proposed exercises (4.5/5). Additionally, only three patients found the acquisition session a bit hard (4/5), while it was generally considered adequate (1.7/5).

### The collaborative model: actions taken and results

#### Design

The assessment of a collaborative model between researchers and POs considered several aspects since the beginning, including the available literature on the topic, the expectations of PO and researchers, the project goals and timelines, and the expertise involved in the use case.

The actions taken during the use case stages were derived from the concoction of an expert team and PO points of view. Initially, researchers presented the project and its intended use case to As.It.O.I. representatives, providing them with a clear overview of the scientific concept in several meeting sessions and helping them to understand the nature and the scope of the study.

The second step was to draft the technical protocol to which a team of experts, with extensive experience in OI, comprising clinicians, engineers, biologists, psychologist, and physiotherapists, contributed. This protocol defined the general terms of the experimental procedure for the fully instrumented movement analysis, including the motor tasks to be tracked and the instrumentation to be used, taking into consideration also the use case aims, the feasibility aspects (i.e. time and efforts necessary for data collection) and the potential impact on OI patients from the perspective of healthcare providers and researchers. The experimental protocol also specified aspects related to data collection in the ROI registry, the tools for evaluating the HRQoL of patients as well as the questionnaires for measuring their confidence during daily activities. The draft was shared with As.It.O.I. to review and to identify potential barriers. This allowed for the incorporation of a number of patient suggestions and perspectives throughout the entire document. The joint evaluation resulted in a feasibility study, which assessed use case endpoints in terms of exercises, timeline, sample size, ambulation level and related issues, age range at recruitment, and OI type. As.It.O.I. helped the researchers to identify potential limitations and, by working together, solutions to overcome or to minimize them were found. According to the literature, it is considered ethically beneficial to incorporate patients’ perspectives into research protocols and documentation [13, 25, 33]. Our experience aligns with this, as the PO’s engagement in drafting and refining the protocol proved valuable. In confirmation of the hypothesis that patient-centred research studies are more favourably considered by ethics committees (EC) [25], the protocol for the present study received expeditious and complete approval without the need for any additions or modifications.

#### Planning

The open dialogue with the PO facilitated the creation of a shorter and layman version of the protocol (a one-page document) to be shared with patients and their families, thus promoting the dissemination of the study, its scopes, and the experimental procedures. This document succinctly describes the motor tasks to be performed and includes some practical tips for the participants. The primary objective of this document was to reach the patients providing them with clear and comprehensive information, avoiding scientific jargon while still maintaining the appropriate content, thus overcoming the complexity of the technical language and protocols.

#### Conduct and dissemination

The As.It.O.I. supported and promoted the dissemination of the use case through its channels. The most effective method was at their annual meeting. In fact, in May 2023, we were invited by the PO to present the ongoing study to patients and their families, allowing the audience to raise any questions and to address any potential doubts. By stimulating patients’ interest and clearly explaining the study goals, PO helped us in actively promoting the recruitment of participants. In fact, after the meeting, several patients contacted the use case staff for self-proposing as potential candidate for the study. Additionally, a dedicated project page was created on As.It.O.I. website to spread the information, further promoting layman dissemination [34].

The POs have a recognized role also in the dissemination activities of findings, particularly beyond the scientific audience [13, 14, 26] which is addressed carefully by the researchers involved in the study. Our experience echoes the literature, since this collaboration allows us to broaden the dissemination of the study results, with particular attention to plain language dissemination. Additionally, in terms of results and dissemination, a last aspect worth mentioning is the following event: the As.It.O.I. president presented a dissertation at the “Patient Expert Training for Digital Health Technologies” at the “Unitelma Sapienza” University of Rome, titled “Evaluation of movement in Osteogenesis Imperfecta patients via gait analysis: opportunities and challenges for patient expert” as master thesis exactly focused on this use case.

The expected financial barriers and the economic impact on patients who are interested in taking part in a clinical research study represent an undoubtedly critical aspect of the study itself. In our experience, the PO also faced this aspect and supported the study feasibility, by providing financial support for patients. In particular, As.It.O.I. reimbursed PO members who decided to take part in the use case. The reimbursement consisted of an economic token structured in three levels, with a graduation of economic contribution dependent on the distance from our Institution. In the case of children, or when otherwise appropriate, the reimbursement included the financial support also for a caregiver. This enables PO to evolve into genuine strategic contributors within the realm of clinical development.

Overall, the As.It.O.I. involvement greatly contributed to the feasibility and success of the study, despite the constrained timeframe. This contribution was achieved through the promotion of patient engagement, the establishment of additional dissemination channels, and the provision of financial assistance to patients.

## Discussion

### Lesson learned

**Fig. 2:**
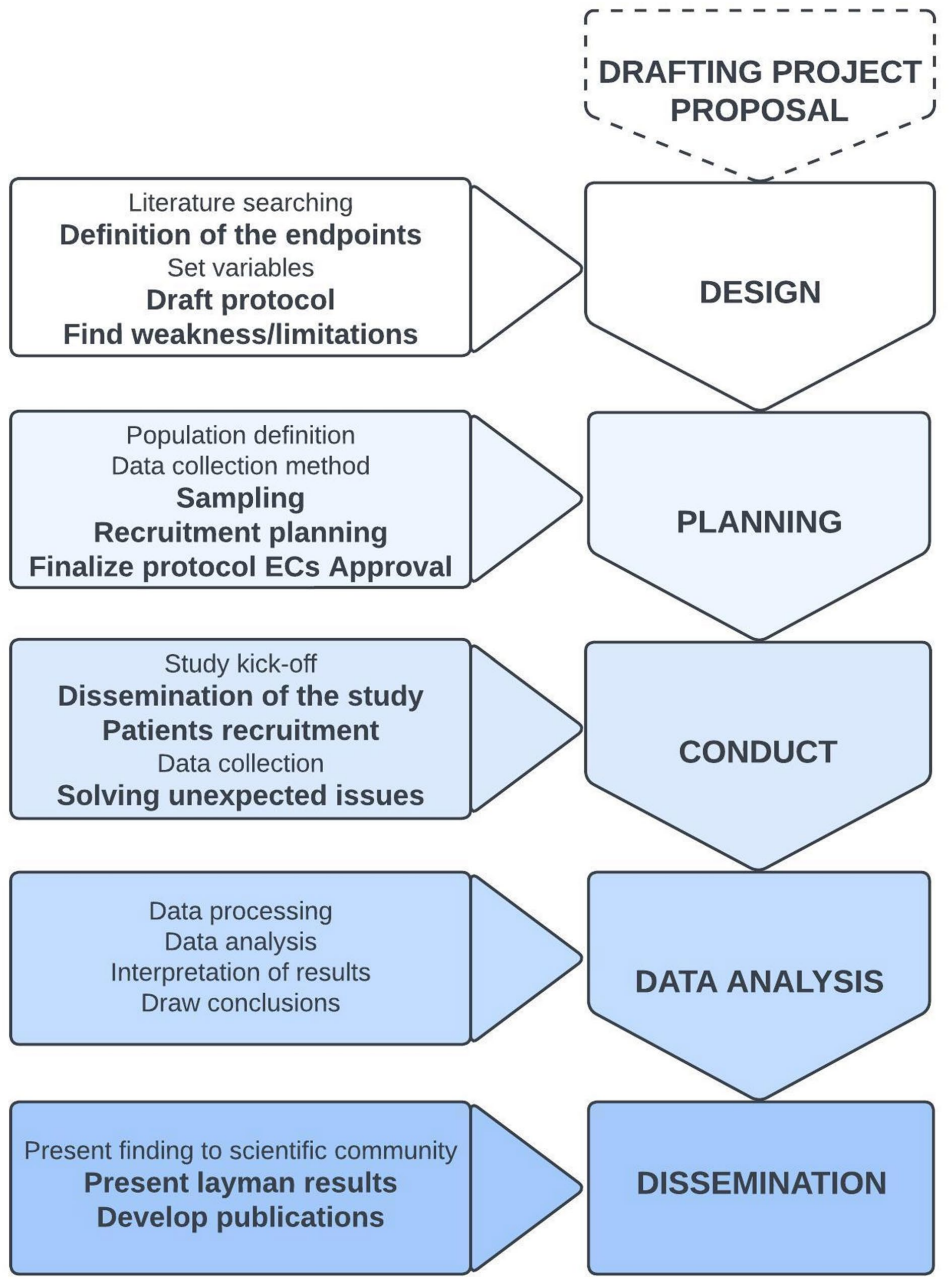
The research stages and the PO involvement. The different sequential stages of the present research study: on the right there are the five research stages; on the left there are the corresponding main research activities; here in bold text, the actions and activities that gain benefit from the PO involvement

The topic of how POs experience research studies and collaborate with researchers is an ongoing subject of debate [27]. The POs’ role is expanding, and their involvement may take various forms, often resulting in an almost unique dynamic for each clinical research study [35]. The distribution of actions and activities, integration, and combination of different expertise, as well as the definition of roles, can sometimes be blurred and challenging in the interaction between POs and researchers. However, we have not encountered such issues in the present experience, which is likely due to the long-standing relationship and the mutual respect that has been fostered over the years between IOR and As.It.O.I. To generalize our collaborative experience, we have individuated which steps and stages of the research process can benefit the most from the PO support (Fig. 2). We have further identified several gains that can leverage the research studies in disparate ways; on the other hand, those weaknesses which may impede or delay the valuable results of this approach have been identified.

#### Advantages

Among the several concrete and measurable results explicitly related to this specific use case, there are generic additional benefits that positively impact research studies and potentially clinical trials.

Indeed, the involvement of patients with rare and ultra-rare diseases in the early stages is pivotal to maximize the chance of a success for the clinical trial. In fact, POs can help to identify some of the undesired design aspects that could be modified or removed to make the trial more acceptable for potential participants [36]. One of the main challenges is represented by the difficulty in clearly determining and agreeing on which outcome should be measured since the health outcomes (i.e. symptoms and signs of the disease) can be very different across individuals and these can change as the medical condition progresses [37]. The collaborative model between POs and researchers improves the research studies, allowing patients to make their voices heard and increasing the patient-centricity of outcomes and endpoints, as per the literature [6]. This aspect emerged clearly from the satisfaction questionnaire results showing that the interaction between researchers and PO allows to promote proper study information and to define a feasible set of exercises. Through listening and combining member experience, POs have an invaluable function because these are capable of developing a collective knowledge that is, notwithstanding differences, just as effective as that of the researchers. The first-hand experience with the disease can offer different inputs and perspectives for the study and frequently can help in proposing different effective solutions.

The combination of both perspectives and the derived collaborative approach incentivizes researchers to engage in better documentation and research methods, resulting in a positive impact on the approval of the ethical committee. In fact, POs’ review of the relevant consent forms, protocols and other study documentation helps to produce more participant-friendly documents, which are positively evaluated by ECs, as well as to influence the recruitment phase and to reduce participant withdrawals [38]. The POs are uniquely positioned to provide objective and comprehensive evaluations of the consent forms and protocol for patients. By following their guidance, researchers can produce more concise and understandable documentation for patients, ensuring a clear understanding of the study and its necessary requirements.

Additionally, an increased participation of POs in the field of clinical trials has also been recognized as effective by the Italian government as per Regulation (EU) No. 536/2014, (nationally approved as L. 11/01/2018, Art. 1) which guarantees the preference to centres that ensure, in phase IV, the involvement of POs in the definition of the research protocols, particularly for RDs [39].

Financial aspects, in terms of co-financing research activities, have historically been one of the primary reasons for the involvement of patient organisations in research studies and clinical trials, in addition to the mere provision of biosamples [19, 35]. Although this role is still an important part of the research process, the POs should not be seen as mere token providers. On the contrary, to date in several European-funded projects and programmes, like Innovative Medicine Initiative, POs participation is encouraged, and they are eligible to directly receive funding, considering their role in providing beneficial insights [40]. Therefore, POs could be actively part of the research process, beginning from proposal drafting and fundraising.

One of the most acknowledged aspects of POs involvement is the dissemination of the study before, during and after its completion. Literature is primarily focused on the dissemination of research findings [13, 19] while in our experience, POs can play a pivotal role also before and during the studies. POs are capable of implementing several actions in a different manner -distinct from researcher ones- in terms of target audience and communication style. POs typically transfer knowledge using patient-friendly language and alternative channels of communication, in comparison with the scientific community. In fact, POs frequently use their websites and social media to reach a lay audience, as well as patients’ meeting, succeeding in spreading research objectives and findings to interested patients. The joint disseminating effort allows to promote research studies and to diffuse the results on a broader and more inclusive scale.

Another impacting advantage is the development of a stronger relationship between public institutions and POs. As previously shown, this approach can facilitate ongoing studies and above all can pave a way of trust for future clinical trials and research collaborations. Such interactions may also lead to wider cooperation (i.e. public-private partnerships) aiming at increasing the output and productivity of multiple stakeholders’ efforts. Besides, this collaborative model grants to design and to promote patient-centred studies and trials with a more holistic approach, particularly for RDs field and in vulnerable patients’ scenarios.

Including the patient voice and involving POs from the earliest stages of research, such as fundraising activities and drafting project proposals, increases the success rate of the project [38]. This contribution is considered so much influential that the European Commission encourages the participation of patients’ organisations in EU-funded health projects. The European Commission also emphasizes that public institutions and POs collaboration is crucial to attaining truly patient-centred outcomes and highly effective projects.

#### Disadvantages

The first potential limitation that a collaborative approach and an open dialogue between POs and researchers may face is a lack of knowledge of the former on specific topics [38]. POs are sometimes unaware of specific processes and procedures and unfamiliar with terms and terminology related to clinical trials and European programmes. Nonetheless, an increasing number of patients are currently overcoming this limitation through training provided by programmes such as the European Patients’ Academy on Therapeutic Innovation [EUPATI - https://eupati.eu/] which aimed to “increase the capacity and capability of patients and patient representatives to understand and meaningfully contribute to medicines research and development, and to improve the availability of medical information for patients and other stakeholders”.

Involving POs can occasionally cause delays in research timelines, due to the need for additional moments of confrontation, revision of research protocols, preparation of additional documents and related activities. Research studies require addressing several complex tasks, and actions cannot be improvised. Although we have experienced an increase in the time needed to align POs and researchers as well as to revise the documents, the downstream benefits were much more valuable in terms of more efficient activities and overall time reduction.

As mentioned, significant heterogeneity exists among different POs due to various factors such as size, age, goals, and financial resources [13, 15]. POs may also differ on their home country, development over time, level of operation (national, European, international) and type of organisation (such as associations for social promotion, non-profit organisations, etc.), and the related normative framework that regulates their activities. Therefore, the involvement of different POs may lead to varying processes and outcomes.

As a matter of fact, the nature of this type of collaboration requires an open-minded approach. This may prove challenging for researchers who are accustomed to collaborating solely, or primarily, with other researchers and clinicians and may not be inclined to fully consider the patient’s perspective and to listen to their voice.

Bilateral proactive actions and clear bidirectional communication can resolve several of these disadvantages, reducing or mitigating the described barriers. Nevertheless, guidelines for collaboration may also help in overcoming these limits [28]. As an example, the Value + project has produced a toolkit titled “The Value + Toolkit. For Patient Organisations On Meaningful Patient Involvement: Patients Adding Value To Policy, Projects And Services” [41]. This toolkit highlights the active role of POs in decision-making and proposes good practices for their involvement in European projects. Similar guidelines and indications may simplify and regulate the collaboration between POs, researchers and other potential stakeholders, leading to even better research studies.

Finally, we have to mention a limitation encountered during the project. In fact, the 4FRAILTY project approval faced some bureaucratic issues, causing a considerable general delay in the starting of the research activities, which has shortened considerably the timeframe of the project and, consequently, the use case duration. This delay had negatively impacted on the research study as thus the patients’ enrolment was only open for 10 months.

## Conclusion

The intervention of patient organisations is becoming increasingly significant in clinical research studies, as well as in clinical trials and projects. This experience resulted in several benefits to all actors involved, reinforcing the collaboration between the PO and researchers, and fostering a cohesive and cooperative network that supported effective enrolment, optimized study design and documentation and improved dissemination strategies. Collaboration based on mutual understanding and learning is the foundation of an innovative concept: moving from “research for patients” to “research with patients”.

By the strengthening of a more collaborative involvement of patient organisations, it is possible to maximise the research process and to facilitate patient-centred outcomes, while providing the best quality of information to patients with rare diseases.

## Data Availability

Not applicable.

## Abbreviations

4FRAILTY: Advanced solutions for the management of patients with Osteogenesis Imperfecta project
ABC Scale: Activities-specific Balance Confidence Scale
As.It.O.I.: Italian Patients Association of Osteogenesis Imperfecta
EC: ethics committees
ERNs: European Reference Networks
EQ-5D: EuroQoL-5-Dimensions
EUPATI: European Patients’ Academy on Therapeutic Innovation
EURORDIS: European Organisation for Rare Diseases
HRQoL: Health-related quality of life
IMU: inertial measurement unit
IOR: IRCCS Istituto Ortopedico Rizzoli
OI: Osteogenesis Imperfecta
OIFE: Osteogenesis Imperfecta Federation Europe
POs: patient organisations
RDs: Rare Diseases
ROI: Registry of Osteogenesis Imperfecta
